# Downregulation of *RBBP6* variant 1 during arsenic trioxide-mediated cell cycle arrest and curcumin-induced apoptosis in MCF-7 breast cancer cells

**DOI:** 10.2144/fsoa-2019-0047

**Published:** 2019-07-30

**Authors:** Lilian Makgoo, Kagiso Laka, Zukile Mbita

**Affiliations:** 1 Department of Biochemistry, Microbiology, & Biotechnology, University of Limpopo, Private Bag x1106, Sovenga, 0727, Polokwane, South Africa

**Keywords:** apoptosis, arsenic trioxide, breast cancer, G2/M arrest, *RBBP6*, splicing

## Abstract

**Aim:**

To determine the expression patterns of the *RBBP6* spliced variants during arsenic trioxide-mediated cell cycle arrest and curcumin-induced apoptosis in MCF-7 cells.

**Materials & methods:**

As_2_O_3_ and curcumin were used to study cytotoxicity, cell cycle arrest, apoptosis and the expression of *RBBP6* variants. The MUSE Cell Analyser was used to analyze cell cycle arrest, apoptosis and multicaspase activity while apoptosis was further confirmed using microscopy. Semi-quantitative RT-PCR was employed to quantitate the expression of the *RBBP6* variants.

**Results:**

This study showed that the MCF-7 cells expressed *RBBP6* variant 1 but lacked both variant 2 and variant 3. Both As_2_O_3_ and curcumin significantly downregulated *RBBP6* variant 1 (p < 0.001).

**Conclusion:**

*RBBP6* variants are promising therapeutic targets.

## Lay abstract

Understanding how breast cancer develops remains to be fully understood. It is known that genes play a pivotal role in the carcinogenesis process. The *RBBP6* gene, which has different variants, has been reported to be involved in cancer development but it remains unclear which *RBBP6* product is involved in cancer development. This study has demonstrated that there are *RBBP6* variants that are pro-carcinogenic and those that are anti-carcinogenic. This study further showed that arsenic trioxide and curcumin lowered the expression of the *RBBP6* splice variant 1 in MCF-7 breast cancer cells. Therefore, targeting *RBBP6* variants for future drug development is a promising strategy.

Cancer is an uncontrollable growth of cells due to loss of cell growth regulation, which normally results in high proliferation rates, resistance to apoptosis and activation of survival pathways. Breast cancer is no exception to this trend and is the most aggressive and leading cause of death among woman, worldwide [1]. Despite the progress made in the improvement of breast cancer treatment strategies, it is still difficult to destroy breast cancer cells without affecting the normal cells and minimize the long- and short-term side effects associated with the current breast cancer treatment [2].

Gene therapy has attracted a lot of interest for breast cancer treatment. The search for regulatory biomolecules involved in the carcinogenesis process has also intensified. *RBBP6* is one of the genes that has shown great potential as therapeutic targets [3–5]. RBBP6 is a protein encoded by the *RBBP6* gene located in chromosome 16p12.2. In humans, the *RBBP6* gene is transcribed into three proven spliced variants due to alternative splicing [4]. However, Ntwasa *et al*. [6]. reported the existence of a fourth variant due to alternative splicing. These alternatively spliced variants include a 6.1-kb mRNA transcript and a 1.1-kb mRNA transcript (variant 3). The 6.1 kb variant is further alternatively spliced into two mRNA variants, one containing exon 16 (variant 1) while the other lacks exon 16 (variant 2), consequently resulting in three mRNA transcripts [4]. Variant 3 is translated into RBBP6 isoform 3. Isoform 1 is made of 1792 amino acids, followed by isoform 2 with 1758, and last isoform 3 with 118 amino acids [4]. Recently, it was reported that there are four isoforms but it is not clear how the fourth isoform is derived. The hypothetical isoform 4 has been reported to have 952 amino acids [7] and its function remains a mystery.

Little is known about the expression and regulation of human *RBBP6* splice variants during cell cycle progression and breast cancer development. Previously, one study had reported the involvement of the RBBP6 rat homolog [8], proliferation potential protein-related (P2P-R), in mitotic apoptosis. It is only Mbita *et al.* that implicated the human RBBP6 in cell cycle regulation [4]. Recently, another study reviewed the role of *RBBP6* in carcinogenesis, implicating *RBBP6* as a key role player in both carcinogenesis and cell cycle regulation [7].

The expression of the *RBBP6* transcripts is not fully understood, especially during cell cycle arrest and apoptosis in breast cancer. This study explores the possible involvement of *RBBP6* transcripts in breast cancer development and their roles in the regulation of cell cycle and apoptosis. In this paper, the analysis of *RBBP6* transcripts during cell cycle arrest and apoptosis was carried out to assess the possible involvement of *RBBP6* variants in the carcinogenesis process, using MCF-7 breast cancer cells. Deregulation of both cell cycle and apoptosis are some of the manifestations of the carcinogenesis process. Arsenic trioxide and curcumin have been shown to induce cell cycle arrest and apoptosis while cobalt chloride has been demonstrated to induce cell cycle arrest [9–11]. The latter was an appropriate cell cycle arrest positive control [11]. The roles of arsenic trioxide and curcumin in cell cycle arrest and apoptosis induction are well understood. Both cytotoxic agents were therefore suitable for studying the expression of *RBBP6* transcripts during cell cycle arrest and apoptosis of MCF-7 breast cancer cells.

## Materials & methods

### Cell lines, cell culture maintenance & reagents

The breast cells, MCF-7 (ATCC-HTB-22, biosafety level II), were kindly donated by Prof Mervin Meyer from the University of the Western Cape who had purchased the cells from the American Type Culture Collection (ATCC, VA, USA). The MCF-7 cells were cultured in complete medium (Dulbecco’s Modified Eagle's Medium [DMEM] supplemented with 10% fetal bovine serum [FBS] and 1% penicillin–streptomycin–neomycin) in a humidified atmosphere of 5% CO_2_ at 37°C_._ Curcumin, arsenic trioxide, cobalt chloride and MTT [3-(4, 5-dimethythiazol- 2-yl)-2, 5-diphenyltetrazolium bromide] were bought from Sigma-Aldrich (South Africa).

### MTT assay

The effect of arsenic trioxide on the growth and viability of MCF-7 cells was assessed using the MTT assay. Briefly, breast cancer cells (MCF-7) were seeded in a 96-well microtiter plate at 4 × 10^3^ cells/well and exposed to various concentrations of As_2_O_3_ (0–64 μM/ml) for 24 h after allowing them to attach, overnight. After 24 h, the treatment was removed followed by addition of 10 μl of MTT (5 mg/ml) into each well and an additional 4-h incubation at 37°C. An MTT solution was aspirated off and, 100 μl of DMSO was added into each well to achieve solubilization of the formazan crystals formed in viable cells before absorbance at 570 nM could be measured, using a GloMax-Multi+ (Promega, WI, USA). Cell survival rate was calculated using the following formula:

$$Survival\;rate\;(\%)=\frac{Average\;OD\;(experimental\;group)}{Average\;OD\;(Untreated\;group)}\times 100\%$$

The MTT assay was also performed for the positive control (cobalt chloride).

### Morphological assessment of apoptosis using DAPI staining & light microscopy

DAPI staining was used for the morphological evaluation of MCF-7 cells after the treatment with arsenic trioxide and curcumin, as well the positive control (cobalt chloride). Briefly, MCF-7 cells were plated at a density of 4 × 10^3^ cells per well in a six-well plate in a complete medium for 24 h and starved for further 12 h for synchronization. The cells were treated with arsenic trioxide, curcumin and cobalt chloride for 24 h. After incubation, the cells were washed with 1× PBS and stained with 5 μg/ml DAPI for morphologic assessment of apoptosis using the Nikon Eclipse TS100F Ti-E inverted microscope (Nikon Instruments, Shinagawa, Japan).

### Cell cycle arrest analysis

The MCF-7 cells were plated at a density of 4 × 10^3^ cells per T-25 culture flask in a complete medium for 24 h, and further starved for 12 h to synchronize the cells. After 24-h treatment with IC_50_s for arsenic trioxide and the positive control (cobalt chloride), the relative number of cells in distinct cell cycle phases were assessed using the Muse™ Cell Cycle Kit following the manufacturer’s instructions (Merck Millipore, Darmstadt, Germany) and analyzed using the Muse^®^ Cell Cycle Analyzer (Merck Millipore).

### Multi-caspase assay

The MCF-7 cells were cultured in 6-well plates, overnight; starved for 12 h and treated for 24 h with curcumin, which also served as a positive control and arsenic trioxide concentrations (11 and 32 μM). After treatment with the arsenic trioxide and the positive control (curcumin), the percentages of cells with activated caspases were quantified using the Muse™ Multi-Caspase kit following the manufacturer’s instructions (Merck Millipore) and analyzed using the Muse Cell Analyzer.

### Apoptosis assay

The MCF-7 cells were cultured in 6-well plates and allowed to settle overnight; starved for 12 h and then treated for 24 h with curcumin, which also served as a positive control and arsenic trioxide concentrations (11 and 32 μM). After treatment with arsenic trioxide and curcumin, the percentages of apoptotic cells were quantified using the Annexin V/FITC kit following the manufacturer's instructions (Merck Millipore) and analyzed using the Muse Cell Analyzer.

### Semi-quantitative reverse transcription polymerase chain reaction

The MCF-7 cells were plated at a density of 4 × 10^3^ cells per T-25 culture flask in complete medium for 24 h and starved for further 12 h. The cells were then treated with arsenic trioxide, cobalt chloride and curcumin and incubated for 24 h. The untreated control and the treated samples were washed with 1× PBS, twice. Total RNA from all the cell groups was extracted using the TRIzol reagent (Thermo Fisher Scientific, MA, USA) and reverse transcription (RT) was performed using the AMV reverse transcription system manufacturer’s instructions (Promega, WI, USA). cDNA was amplified using both the *GAPDH* primers (forward primer: AGCTGAACGGGAAGCTCACT; reverse primer: TGCTGTAGCCAAATTCGTTG) and *RBBP6* primer sets specific to different variants:

(*RBBP6* variant 3 – forward primer: GGATAATATGTGGCATCACTTG; reverse primer: TCCCTGTATGACACTGTGTTG and *RBBP6* variant 1 and 2 – forward primer: GTATAGTGTCCCTCCTCCAGG; reverse primer: GTAATTGCGGCTCTTGCCTCT). The PCR reactions were prepared using a 2× PCR Master Mix (Takara Bio Inc., Kusatsu Japan) according to the manufacturer's instructions. The reactions were subjected to 30 cycles comprising the three PCR steps (denaturation, annealing and extension) in the T100 Thermal Cycler (Bio-Rad, CA, USA). The products were electrophoresed on 1% agarose gels using a 100 bp DNA molecular weight marker (BioLabs, MA, USA) to confirm the sizes of the PCR products.

### Immunocytochemistry assay

To evaluate the effect of arsenic trioxide on the expression and localization of Bax protein, immunofluorescence staining was performed. Breast cancer cells (MCF-7) were seeded on cover slips in 6-well plates at 1 × 10^5^ cells/well and exposed to various concentrations of As_2_O_3_ (11 and 32 μM), cobalt chlorite (100 μM) and curcumin (100 μM) for 24 h. After the 24-h incubation, the cells were washed twice with 1× PBS and fixed in 4% paraformaldehyde for 15 min at room temperature. After fixing, the cells were permeabilized with 0.25% Triton™ X-100 for 10 min at room temperature. Antibody-nonspecific binding was blocked using 0.5% BSA for 1 h at room temperature. Following blocking, the cells were incubated with anti-Bax primary antibody (Lot# E0707; Santa Cruz Biotechnology, TX, USA). A 1:50 dilution of the primary antibody was prepared in 1X PBS-BSA (0.5%) and incubated for one hr at room temperature. The cells were washed with sterile 1× PBS, then labeled with Flour^®^ 488 goat anti-mouse IgG secondary antibody (Lot# 1810918; Thermo Fisher Scientific, MA, USA). The cells were counter-stained with DAPI (5 μg/ml) for 10 min and examined under the Nikon Eclipse TS100F Ti-E inverted microscope (Nikon Instruments, Shinagawa Japan).

### Statistical analysis

The results of the different experiments (cell viability assay, cell cycle phases analysis and apoptosis analysis) performed in triplicates were expressed as the mean ± standard deviation (SD) using Instat Version 3 statistical software. Data were analyzed to get statistical significant differences by comparing two datasets (the untreated samples were compared with the treated samples) and the differences were considered significant when *p was ≤ 0.05, **p ≤ 0.01 and ***p ≤ 0.0001.

## Results

### *In vitro* inhibition of MCF-7 cell growth by arsenic trioxide

**Figure 1.**
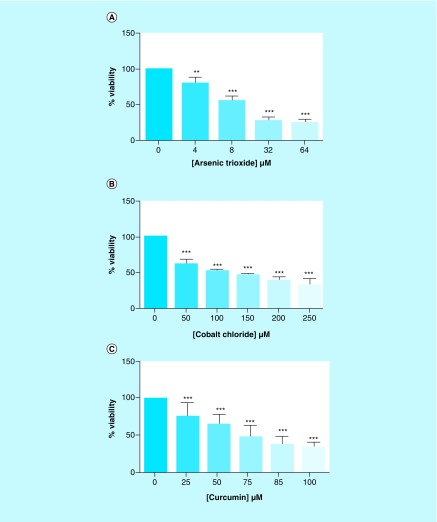
Cell viability analysis of the effect of arsenic trioxide, cobalt chloride and curcumin on MCF-7 cells. **(A)** The effect of arsenic trioxide [As_2_O_3_], **(B)** cobalt chloride [CoCl_2_] and **(C)** curcumin on the cell growth and viability of the human breast cancer MCF-7 cells. All the three compounds inhibited viability of MCF-7 cells *in vitro* when compared with the untreated control cells. Results were obtained from three independent experiments and were presented as ± standard error of the mean and the differences were considered significant when **p ≤ 0.01 and ***p ≤ 0.0001.

The effect of arsenic trioxide on the growth and viability of MCF-7 cells was assessed using MTT assay. As shown in Figure 1A, the arsenic trioxide significantly inhibited the growth and viability of MCF-7 cells in a dose-dependent manner, with the extrapolated IC_50_ of 11 μM and apoptosis-inducing concentration of 32 μM after 24-h treatment. After 24-h incubation, cobalt chloride (positive control for cell cycle analysis) also inhibited cell growth of MCF-7 cells at an IC_50_ of 100 μM (Figure 1B). After 24-h incubation, curcumin, which was also used as a positive control for apoptosis assay, reduced the growth of MCF-7 cells between 85 and 100 μM concentration (Figure 1C). These results demonstrated that arsenic trioxide was potent in inhibiting the proliferation and inducing death of MCF-7 cells *in vitro*.

### Morphological changes of MCF-7 cells due to cell cycle arrest & apoptosis

**Figure 2.**
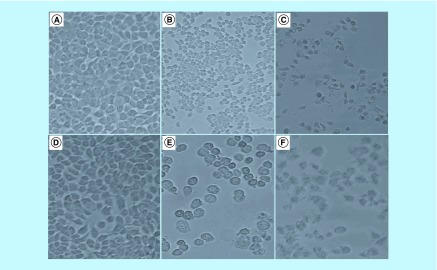
Light microscopy analysis of the effect of arsenic trioxide, cobalt chloride and curcumin on the morphology of MCF-7 cells. Morphological characteristics of MCF-7 cells after the treatment for 24 h with **(2B)** 11 μM and **(2E)** 32 μM arsenic trioxide, **(2C)** 100 μM cobalt chloride and (2F) 100 μM curcumin as compared with **(2A & 2D)** untreated cells.

The treatment of the MCF-7 cells with arsenic trioxide, cobalt chloride and curcumin reduced the MCF-7 cell growth and adherence compared with the untreated control cells. Using DAPI staining and the inverted phase-contrast microscopy, 11 and 32 μM arsenic trioxide, 100 μM curcumin and 100 μM cobalt chloride affected the morphology of the MCF-7 cells exposed for 24 h. Many cells, consequently, shrunk, which is typical of cells undergoing apoptosis and evidently, losing their epithelial morphology (Figure 2A–F).

**Figure 3.**
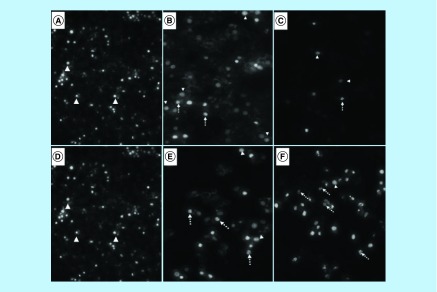
Fluorescence microscopy analysis of the effect of arsenic trioxide, cobalt chloride and curcumin on the morphology of MCF-7 cells. Analysis of morphological effects of the cell cycle and apoptosis inducers, arsenic trioxide, cobalt chloride and curcumin on MCF-7 cells. DAPI staining showed an increase in the mitotic cells (white unbroken arrow) and the apoptotic cells (white dotted arrow) after **(B & E)** arsenic trioxide, **(C)** cobalt chloride and **(F)** curcumin treatments compared with untreated cells (**3A** and **D** with white arrow heads).

DAPI staining revealed that treatment with arsenic trioxide (11 and 32 μM) and the positive controls (cobalt chloride and curcumin) induced typical apoptotic morphology. These included nuclear condensation, lost microvillus and apoptotic body formation (Figure 3A–F). The changes confirmed that arsenic trioxide induced typical cytomorphological features of apoptosis in MCF-7 cells. Loss of microvilli, nuclear condensation and fragmentation were observed after the 24-h treatment of MCF-7 cells with arsenic trioxide (11 and 32 μM), curcumin (100 μM) and cobalt chloride (100 μM).

### Arsenic trioxide induces G2/M cell cycle arrest in MCF-7 cells

**Figure 4.**
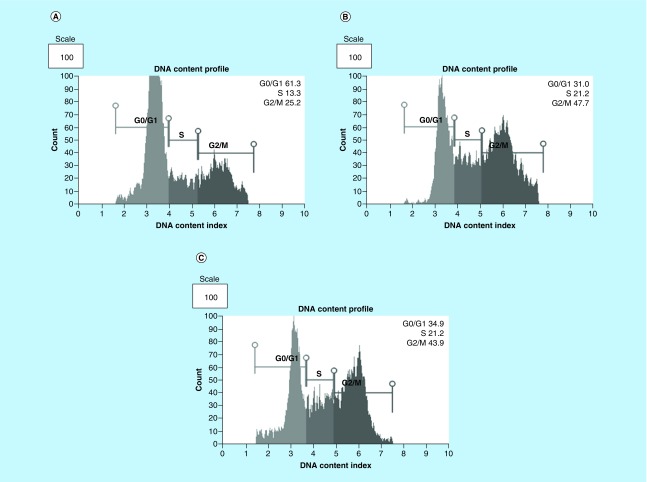
Cell cycle analysis of arsenic trioxide induced G2/M cell cycle arrest in MCF-7 cells. Distribution of MCF-7 cells at distinct cell cycle phases after the treatment with **(4B)** arsenic trioxide and **(4C)** cobalt chloride compared with the untreated cells. Treatment with 11 μM arsenic trioxide and 100 μM of cobalt chloride (positive control) resulted with increased G2/M cell cycle arrest in MCF-7 cells when compared with **(4A)** the untreated control.

**Figure 5.**
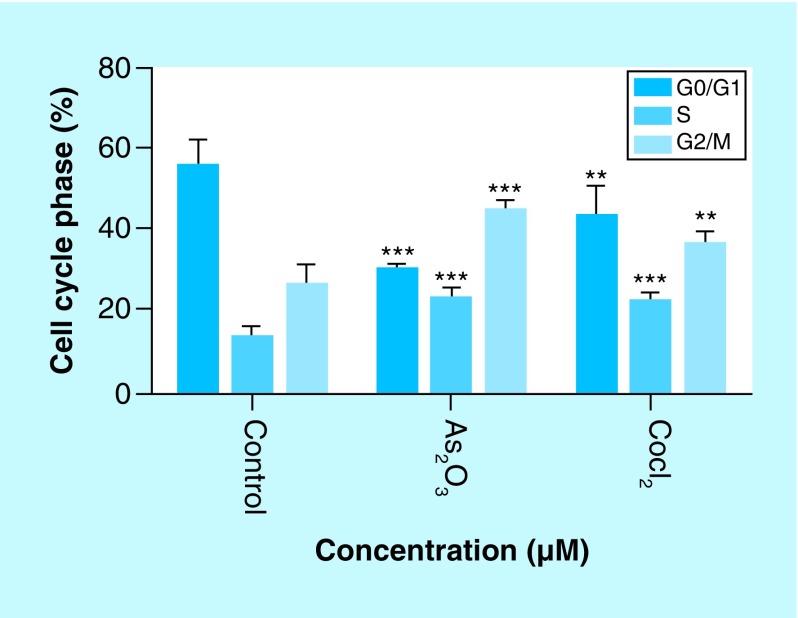
Graphical analysis of arsenic trioxide induced G2/M cell cycle arrest in MCF-7 cells MCF-7 cells. Arsenic trioxide (11 μM) and cobalt chloride (100 μM) induced G2/M arrest relative to the untreated MCF-7 cells. Results were obtained from three independent experiments and were presented as ± SEM and the differences were considered significant when *p was ≤ 0.05, **p ≤ 0.01 and ***p ≤ 0.0001.

The effect of As_2_O_3_ and the cell cycle arrest positive control, cobalt chloride, on MCF-7 cells was observed. Both inducers increased the G2M cell population where the treatment with 11 μM of arsenic trioxide showed an increase in the population of G2/M phase cells after 24 h and decreased the G1 population (Figures 4 & 5) relative to the untreated control. This trend was also observed for the treatment with the positive control, 100 μM cobalt chloride.

### Arsenic trioxide induces caspase-dependent mode of death in MCF-7 cells

**Figure 6.**
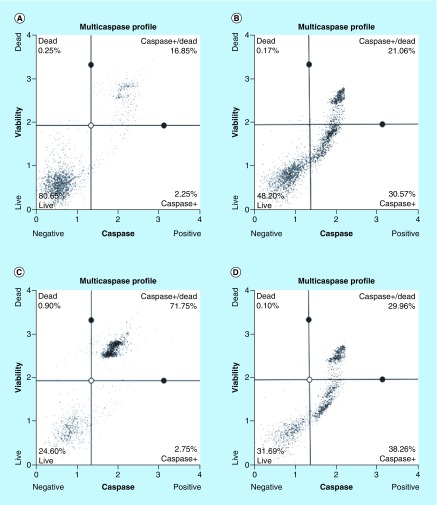
Analysis of arsenic trioxide induced caspase dependent apoptosis in MCF-7 cells. Caspase induction by arsenic trioxide and curcumin in MCF-7 cells. The MUSE^®^ Multi-capsase Analysis confirmed that treatment with 11 and 32 μM of **(B & C)** arsenic trioxide and 100 μM of the positive control, **(D)** curcumin induced caspase-dependent mode of death in MCF-7 cells when compared with **(A)** the untreated control.

**Figure 7.**
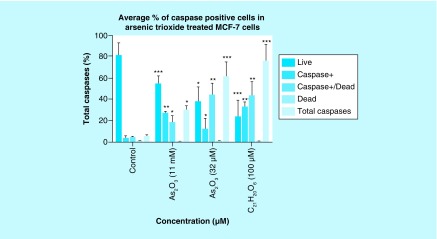
Graphical analysis of arsenic trioxide induced caspase dependent apoptosis in MCF-7 cells. Treatment with arsenic trioxide (11 and 32 μM) and curcumin (100 μM) for 24 h induced caspase-dependent mode of death in MCF-7 cells relative to the untreated control. Results were obtained from three independent experiments and were presented as ± standard error of the mean and the differences were considered significant when *p was ≤ 0.05, **p ≤ 0.01 and ***p ≤ 0.0001.

Caspase activation correlates with the onset of apoptosis and caspase inhibition attenuates apoptosis. Therefore, the involvement of caspases in arsenic trioxide-induced cell death in MCF-7 cells was investigated (Figures 6 & 7). As shown in Figure 6, the percentage of MCF-7 cells undergoing caspase-dependent mode of death after treatment with 11 μM of arsenic trioxide was found to be 51% (Figure 6B). When the concentration of arsenic trioxide was increased to 32 μM, the percentage of cells undergoing caspase-dependent death also increased to 74% (Figure 6C) compared with the untreated control (Figure 6A). The same trend was observed in the positive control (100 μM of curcumin) that showed 67% (Figure 6D).

### Arsenic trioxide induces apoptosis in MCF-7 cells

**Figure 8.**
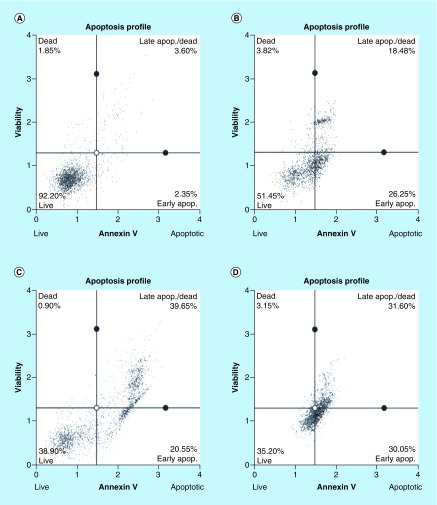
Analysis of arsenic trioxide induced apoptosis in MCF-7 cells. Apoptosis induction by arsenic trioxide and curcumin in MCF-7 cells. The MUSE^®^ Apoptosis Analysis confirmed that treatment with 11 and 32 μM of **(B & C)** arsenic trioxide and 100 μM of the positive control, **(D)** curcumin induced apoptosis in MCF-7 cells when compared with **(A)** the untreated control.

**Figure 9.**
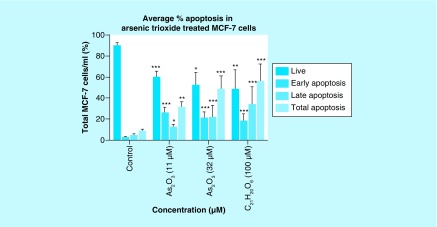
Graphical analysis of arsenic trioxide induced apoptosis in MCF-7 cells. Treatment with arsenic trioxide (11 and 32 μM) and curcumin (100 μM) for 24 h induced apoptosis mode of death in MCF-7 cells relative to the untreated control. Results were obtained from three independent experiments and were presented as ± standard error of the mean and the differences were considered significant when *p was ≤ 0.05, **p ≤ 0.01 and ***p ≤ 0.0001.

To further confirm that arsenic trioxide treatment induced apoptosis in MCF-7 cells, the MUSE^®^ Annexin V analysis was performed. Upon observation under the microscope, MCF-7 cells demonstrated apoptotic changes after treatment with 11 and 32 μM of arsenic trioxide but untreated cells maintained their epithelial morphology and remained attached on the culture flasks. The Muse Annexin V analysis showed that arsenic trioxide remarkably induced apoptosis in MCF-7 cells. As shown in Figures 8 & 9, percentage of cells undergoing apoptosis after treatment with 11 μM of arsenic trioxide was 44% (Figure 8B) and when the concentration of arsenic trioxide was increased to 32 μM, the apoptosis percentage increased to 60% (Figure 8C) when compared with the untreated control (Figure 8A), the same trend was observed in the positive control (100 μM of curcumin) that induced 61% apoptosis (Figure 8D) in MCF-7 cells.

### Arsenic trioxide regulates the expression of *RBBP6* transcripts

**Figure 10.**
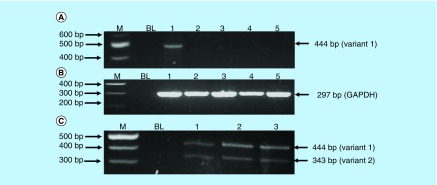
Expression analysis of *RBBP6* variant 1 and 2 in arsenic trioxide treated MCF-7 cells and untreated Hek 293 cells. Amplification of *RBBP6* variants in MCF-7 cells and Hek-293 cells. The primers were designed both upstream and downstream the exon 16 region to amplify both variants 1 and 2. The results **(A)** show untreated MCF-7 cells (lane 1) expressing variant 1, treatment with 11 μM of As_2_O_3_ (lane 2), treatment with 32 μM of As_2_O_3_ (lane 3), treatment with 100 μM cobalt chloride (lane 4) and at last treatment with 100 μM curcumin (lane 5), all showing repressed expression of variant 1. **(B)** (Lanes 1–5) represent *GAPDH* that was used as a loading control. Lanes M stand for the molecular weight marker while lanes BL were blank controls. **(C)** (Lanes 1–3) show the expression of both variants 1 and 2 by the untreated Hek 293 cells.

Messenger RNA (mRNA) levels of *RBBP6* transcripts in MCF-7 cells treated with arsenic trioxide (11 and 32 μM), curcumin (100 μM) and cobalt chloride (100 μM) for 24 h were analyzed using the conventional-PCR to determine whether the *RBBP6* can be implicated in the observed arsenic trioxide-induced cell cycle arrest and apoptosis. The *RBBP6* variant 1 was found to be highly upregulated in the untreated MCF-7 cells when compared with the treated MCF-7 cells. Arsenic trioxide-, cobalt chloride-induced cell cycle arrest and arsenic trioxide- and curcumin-induced apoptosis downregulated the expression of *RBBP6* variant 1. However, these compounds did not induce detectable levels of variant 2 (lanes 2–5 in Figure 10A) nor *RBBP6* variant 4, which remains elusive and undetectable.

**Figure 11.**
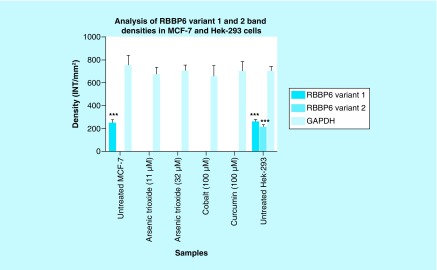
Graphical analysis of *RBBP6* variant 1 and variant 2 band densities in MCF-7 and untreated Hek-293 cells. Untreated MCF-7 cells significantly (***p ≤ 0.001) show increased band intensity of the *RBBP6* variant 1 which diminished after treatment with arsenic trioxide, cobalt chloride and curcumin. Untreated MCF-7 cells did not show any expression of *RBBP6* variant 2, with only untreated Hek-293 cells showing detectable levels of both *RBBP6* variants 1 and 2. Results were obtained from three independent experiments and were presented as ± standard error of the mean and the differences were considered significant when ***p < 0.001. The density was measured using Quanty‐One software.

*GAPDH* was used as a control to ascertain that equal amounts of the cDNAs from untreated and treated samples were used. Figure 10C (lanes 1–3) showed that normal cells (Hek 293 cells) express both variants 1 and 2 with the latter downregulated in breast cancer MCF-7 cells. Treatment of breast cancer cells with both arsenic trioxide and curcumin resulted in downregulation of the *RBBP6* variant 1 in breast cancer cells as shown in Figure 10A (lanes 2, 3, 5), respectively. On the contrary, for the first time, we show that MCF-7 cells either do not express *RBBP6* variant 2 or these cells express this variant at undetectable levels. Our results quantified from three independent experiments using Quantity One^®^ 1D analysis software (Figure 11) showed that variant 1 is highly expressed in MCF-7 cells and is downregulated by apoptosis inducers, arsenic trioxide and curcumin.

**Figure 12.**
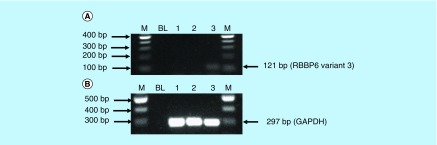
(A & B) Expression analysis of *RBBP6* variant 3 in untreated MCF-7, Caski and Hek-293 cells. Amplification of *RBBP6* variant 3 in MCF-7 cells, Caski cells and Hek 293 cells. The result **(A)** shows untreated MCF-7 cells (lane 1) and untreated Caski cells (lane 2) both with an undetectable expression of *RBBP6* variant 3, only untreated Hek 293 cells (lane 3) show the expression of *RBBP6* variant 3. **(B)** (Lanes 1–3) represent *GAPDH* that was used as a loading control. Lanes M stand for the molecular weight marker while lanes BL were blank controls.

**Figure 13.**
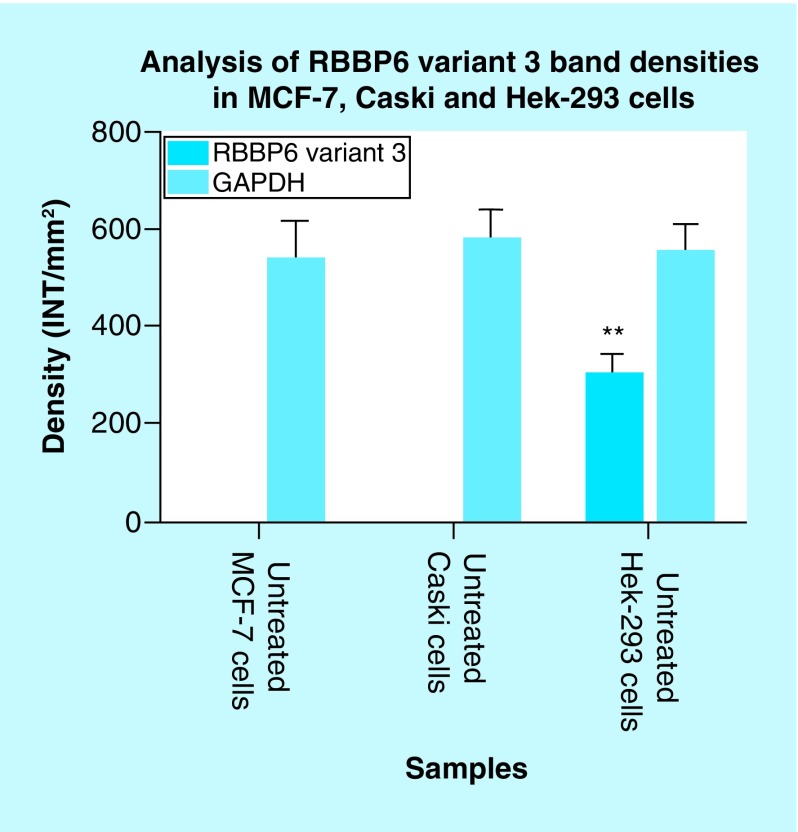
Graphical analysis of *RBBP6* variant 3 band densities in untreated MCF-7, Caski and Hek-293 cells. Both untreated MCF-7 and Caski cells had undetectable expression of *RBBP6* variant 3, only untreated Hek-293 cells show detectable levels of *RBBP6* variant 3. Results were obtained from three independent experiments and were presented as ± standard error of the mean and the differences were considered significant when **p ≤ 0.001 The density was measured using Quanty‐One software.

The mRNA levels of *RBBP6* variant 3 were also determined using conventional-PCR. Figure 12 shows that the *RBBP6* variant 3 is expressed by normal embryonic kidney cells compared with the breast cancer cells. The blank controls (Figure 12, lanes BL) showed no product as expected. The untreated Hek 293 cells (Figure 12A, lane 3) showed expression of the *RBBP6* variant 3 while *RBBP6* variant 3 was undetectable in MCF-7 cells (Figure 12A, lane 1) and in cervical Caski cells (Figure 12A, lane 2). These results were quantified from three independent experiments using Quantity One^®^ 1D analysis software (Figure 13). Our results showed that both variants 2 and 3 were undetectable in cancer cells, especially breast cancer MCF-7 cells and cervical cancer Caski cells. This suggests that both variants 2 and 3 may be crucial in maintaining cell homeostasis, which is lost during carcinogenesis while the expression of variant 1 may favor the carcinogenesis process.

### Arsenic trioxide regulates the expression of Bax protein in MCF-7 cells

**Figure 14.**
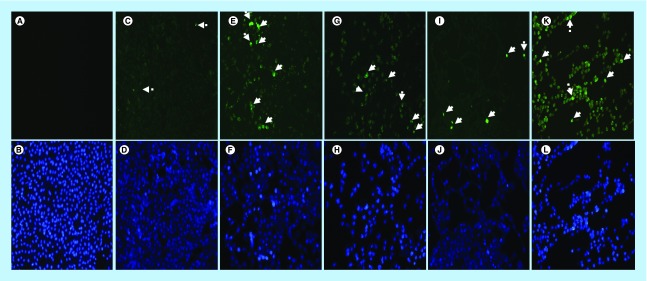
Analysis of Bax localization and expresssion in MCF-7 cells. Localization and expression of Bax in MCF-7 cells counterstained with DAPI. The results show **(A & B)** a negative control, no labeling is seen. **(C–L)** Micrographs show positive Bax staining in the nucleus and cytoplasm. MCF-7 cells show less expression of Bax in **(C & D)** untreated cells because apoptosis is inhibited in these cells, but the treatment with **(E–H)** arsenic trioxide upregulated the expression of this protein. The same trend was observed even in the positive controls, **(I & J)** cobalt chloride and **(K & L)** curcumin which also regulated the expression of Bax protein. White arrows point to mitotic cells with increased Bax staining levels. Magnification 20×.

**Figure 15.**
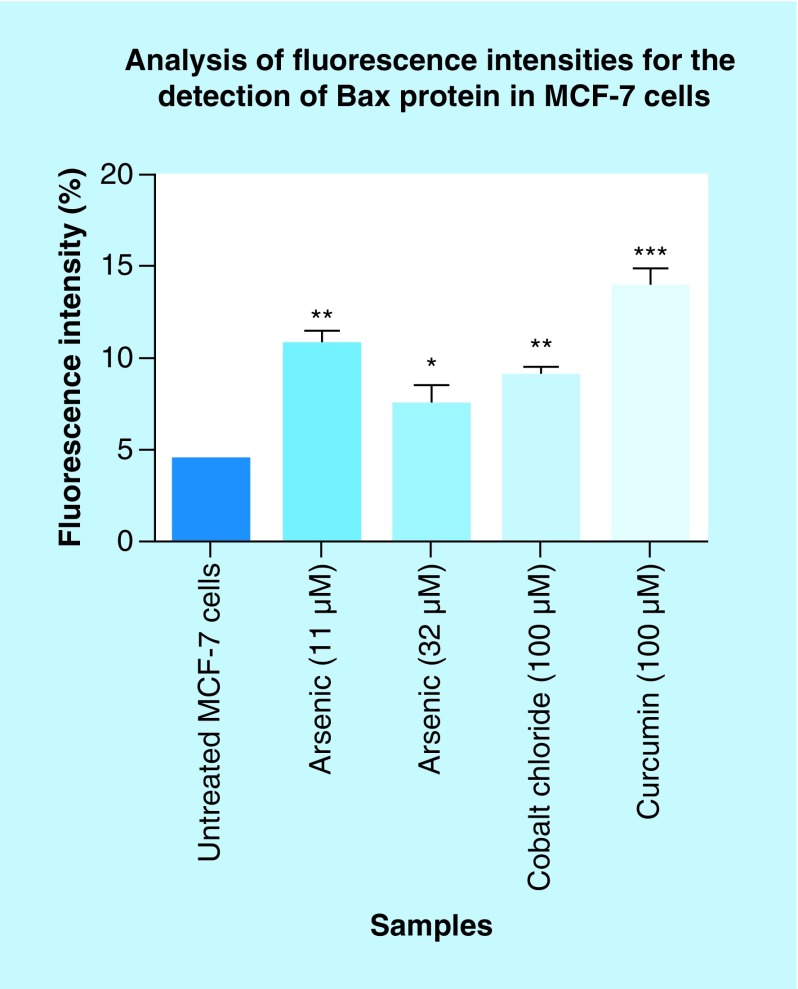
Graphical analysis of fluorescence intensities for the detection of Bax protein in MCF-7 cells. Arsenic trioxide upregulated the expression of Bax protein when compared with the untreated control. The same trend was observed even with the positive controls (cobalt chloride and curcumin). Results were obtained from three independent experiments and were presented as ± standard error of the mean and the differences were considered significant when *p was ≤ 0.05, **p ≤ 0.01 and ***p ≤ 0.0001. The fluorescence intensity was measured using the Image J software (https://imagej.nih.gov/ij/docs/index.html).

In this section, the effect of arsenic trioxide on Bax expression was investigated. Bax protein is lowly expressed in MCF-7 breast cancer cells (Figure 14C & D) because it is involved in the induction of apoptosis, which is a process that is inhibited in breast cancer. The treatment of MCF-7 breast cancer cells with arsenic trioxide showed increased expression of Bax protein (Figure 14E–H). The same trend was observed in curcumin (Figure 14K & L) treated cells while cobalt-induced cell cycle arrest did not have increased significant effect in the expression of Bax (Figure 14I & J). These results (Figure 14 & 15) show that arsenic trioxide is not only inducing apoptosis, cell cycle arrest and regulating the expression of RBBP6 variants but it also upregulates the expression of Bax protein in MCF-7 breast cancer cells, which suggest that *RBBP6* variant 1 downregulation may be involved in the intrinsic apoptotic pathway.

## Discussion

This study showed that *RBBP6* variant 1 may be involved in breast cancer development and its expression in breast cancer cells may promote cell survival. It is not surprising that upon apoptosis induction, this variant is downregulated. On the other hand, the smaller *RBBP6* variant 3 might be involved in the regulation of cell cycle arrest, especially G2/M cell cycle arrest as previously shown in kidney embryonic cells [4], which was undetectable in breast cancer cells. Downregulation of cell cycle regulatory biomolecules favors the carcinogenesis process, hence downregulation of *RBBP6* variant 3 in breast cancer is not surprising. It should be stressed that apoptosis is a well-defined and probably the most frequent form of programmed cell death, but nonapoptotic types of cell death might also be of biological significance [12].

Breast cancer cells are resistant to apoptosis, consequently, growing uncontrollably. In this study, we have shown that breast cancer cells may have lost the expression of *RBBP6* variants 2 and 3 in favor of the carcinogenesis process. *RBBP6* variant 4 has not been demonstrated but it is likely that it is one of the anticancer variants, which may be critical for maintaining cell homeostasis. *RBBP6* has a wide range of functions and these include a role in cell cycle regulation, apoptosis, protein stability and mRNA processing [8,13,14]. *RBBP6* has been shown to induce cell cycle progression by ubiquitinating p53 through Murine Double Minute 2 (MDM2), therefore, promoting carcinogenesis [3]. *RBBP6* variant 1 has ubiquitin ligase activity and this activity leads to enhanced degradation of p53, the cell guardian, which is crucial for antitumor formation. Furthermore, *RBBP6* variant 3 is involved in G2/M arrest, but its absence triggers cell cycle progression and high proliferation rates in normal kidney cells [4]. Consequently, it would be advantageous for cancer cells not to express this variant to support their rapid growth. Some lines of evidence showed that the enhanced expression of different *RBBP6* variants correlate with poor clinical prognosis in colon, prostate and esophageal cancer [15–17]. Breast cancer treatment remains a challenge and therefore, there is a need for more specific and effective therapeutic tools. *RBBP6* is a promising therapeutic target and there are a lot of promising drug development targets, such as arsenic trioxide (As_2_O_3_).

As_2_O_3_ is a potent US FDA approved drug confirmed to treat acute promyelocytic leukemia in patients that relapsed after chemotherapy. Arsenic trioxide exhibits therapeutic effects in a variety of human cancers such as human hepatocellular carcinoma, gastric cancer and cervical cancer [18–20]. Arsenic trioxide hinders cancer development and progression through targeting cellular pathways, leading to inhibition of cell proliferation and invasion, and promoting apoptosis. Little is known about the expression and regulation of the human RBBP6 splice variants by arsenic trioxide during cell cycle progression and breast cancer development. Therefore, in this study, arsenic trioxide was chosen due to its antitumor effect in a variety of human cancers [18–20]. The effect of arsenic trioxide on MCF-7 cells was compared with the positive controls (curcumin and cobalt chloride) because these cytotoxic agents have also been shown to induce cell cycle arrest and apoptosis in cancer cells [10,11]. As previously shown, both the positive controls (curcumin and cobalt chloride) consistently induced apoptosis and cell cycle arrest, respectively in MCF-7 breast cancer cells.

This study has shown that arsenic trioxide and curcumin-induced cell cycle arrest and apoptosis in breast cancer MCF-7 cells, respectively (Figure 1–9). These findings support the results of previous studies showing similar effects on gynecological cancers and other solid tumors [9–11,21]. These findings suggest that As_2_O_3_, cobalt chloride and curcumin should additionally be investigated as potential novel chemotherapeutic agents for the adjuvant treatment of malignant human tumors. It was evident that the untreated control cells retained normal morphology and attached firmly to the culture plates with random orientation (Figure 2), while cells treated with arsenic trioxide, cobalt chloride and curcumin showed remarkable cellular effect, which included decrease in cell numbers, rounding effects, decrease in cell size, detachment from the substratum. Furthermore, DAPI staining (Figure 3B & E) confirmed that arsenic trioxide induced cell cycle arrest and apoptosis that was shown by loss of cell microvilli, nuclear condensation and formation of apoptotic bodies.

Quantitative analysis of apoptotic cells and cell cycle arrest (Figures 4–9) using the MUSE Cell Analyzer demonstrated that As_2_O_3_ induced evident caspase-dependent apoptosis but not necrosis in MCF-7 cells. It was also observed that arsenic trioxide induced G2/M arrest in MCF-7 cells (Figure 4B). The G2/M arrest did not only inhibit the proliferation of cells but also triggered several apoptosis features that were evident after DAPI staining. These results suggest that As_2_O_3_-induced growth inhibition mainly depended on the induction of caspase-dependent apoptosis and cell cycle arrest in MCF-7 cells. These results further showed that caspases are involved in arsenic trioxide induced apoptosis (Figures 6 & 7). They support a study which was done in MCF-7 breast cancer cells to show that As_2_O_3_ exposure significantly increases the level of caspase-3 [22]. The upregulation of caspase 3 by arsenic trioxide was also shown in HT-29 colon cancer cells, suggesting that arsenic trioxide induces caspase-3-dependent pathway [23]. Even though caspase-3 has been previously reported to be inactivated in MCF-7 cells [24], this study has shown that caspase-dependent apoptosis is induced by arsenic trioxide and curcumin and *RBBP6* variant 1 may be critical in the process. Other effector caspases may be involved in the induced apoptosis in MCF-7 cells. There are contradictory reports on the expression of caspase-3 in MCF-7 but it is possible that the mutated form responds to different apoptosis inducers [25].

We further showed that arsenic trioxide and curcumin regulate the expression of *RBBP6* variants, especially the two big transcripts; variants 1 and 2 (Figures 10 & 11). Figure 10A (lane 1) demonstrated that breast cancer cells express *RBBP6* variant 1 and lack the expression of variant 2. Treatment of these cells with arsenic trioxide, cobalt chloride and curcumin diminished the expression of *RBBP6* variant 1. This suggested that this variant may promote breast cancer development while diminished expression of variant 2 may also support carcinogenesis. We previously suggested that *RBBP6* variants and isoforms may have opposing cellular functions [4]. In this study, we showed that *RBBP6* variant 3 is expressed in noncancerous cells, Hek 293 s but undetectable in breast MCF-7 cancer cells (Figures 12 & 13). We also showed that noncancer cells, at least, in Hek 293, express both variants 1 and 2. Arsenic trioxide did not only regulate the expression of *RBBP6* transcripts but also regulated the expression of apoptosis-related protein, Bax (Figure 14). These results showed that As_2_O_3_ is effective against MCF-7 cells and also regulates the expression of *RBBP6* variants, especially variant 1, which makes both *RBBP6* variant 1 and arsenic trioxide an important therapeutic target.

## Conclusion

This study showed that there are *RBBP6* variants that are procarcinogenic and those that are anticarcinogenic. Different *RBBP6* variants could be targeted for cancer therapeutic development. In conjunction with *RBBP6* expression, arsenic trioxide, cobalt chloride and curcumin should be further explored as cancer drugs.

## Future perspective

Little is known about the expression and regulation of the human *RBBP6*-spliced variants by arsenic trioxide, cobalt chloride and curcumin during cell cycle progression and breast cancer development. Taken together our findings indicate that arsenic trioxide, cobalt chloride and curcumin have substantial bioactivity against MCF-7 cells and regulate the expression of *RBBP6* variants. This will allow scientists to target RBBP6 and these cytotoxic agents for future drug development. Further investigations using animal models and other breast cancer types are needed to provide new insights into the potential application of these cytotoxic agents in the treatment of breast cancer and regulation of RBBP6.

**Summary points**

- Arsenic trioxide, curcumin and cobalt chloride decreased the viability of the MCF-7 breast cancer cells in a dose-dependent manner.
- Arsenic trioxide and curcumin-induced, caspase-dependent apoptosis in MCF-7 breast cancer cells.
- Arsenic trioxide and cobalt chloride induced G2/M cell cycle arrest of the MCF-7 cells.
- RBBP6 variants have opposing functions.
- RBBP6 variant 1 may support the carcinogenesis process in breast cancer MCF-7 cells.
- RBBP6 variant 3 is against the carcinogenesis process and it is downregulated in MCF-7 cells.
- Arsenic trioxide upregulated the expression of Bax protein in MCF-7 breast cancer cells.
- Therefore, our study suggests that there are RBBP6 variants that are procarcinogenic and there are those that are anticarcinogenic. It also suggests that arsenic trioxide regulates both RBBP6 transcripts and Bax protein.
